# END-OF-LIFE CARE DURING THE NATIONAL OPIOID CRISIS: A NATIONAL SURVEY OF HOSPICE PROVIDERS

**DOI:** 10.1093/geroni/igz038.3464

**Published:** 2019-11-08

**Authors:** John Cagle, Mary Lynn McPherson, Jodi Frey, Paul Sacco, Orrin Ware, Jack Guralnik

**Affiliations:** 1 University of Maryland, Baltimore, Maryland, United States; 2 University of Maryland, School of Social Work, Baltimore, Maryland, United States; 3 University of Maryland-Baltimore, Baltimore, Maryland, United States; 4 University of Maryland Baltimore School of Medicine, Baltimore, Maryland, United States

## Abstract

No national data exist on drug shortages, missing medications, opioid diversion, and opioid diversion prevention in hospice. We conducted a national survey of hospices (administered June-September, 2018). We randomly selected 600 hospices to survey representatives about: (1) care for patients/families with substance use disorder (SUD) (2) drug shortages; (3) instances of drug diversion; and, (4) drug disposal practices. Surveys were conducted by phone and online. Sample weights were used to adjust for non-response. A total of 371 hospices completed surveys (response rate=62%), 63% of which were administered by phone. Half (50%) of agencies were mid-sized (26-100 patients) and non-profit. Two thirds (66%) of hospices reporting that medications either “never” or “rarely” go missing. On average, there were 0.80 reported cases of confirmed diversion per agency within the past 90 days. Although a majority of hospices (78%) screen patients for SUD, only 43% screen informal caregivers. Just under half (42%) of hospices reported drug shortages over the past year. A minority (8%) of hospices stopped prescribing certain medications altogether due to concerns about diversion. 52% of hospices reported that employees are not allowed to dispose of medications after a home death. Agency representatives estimated that, after a home death, unused opioids were left in the home 32% of the time. On average, hospices have nearly one case of opioid diversion per quarter. Hospices are experiencing medication shortages and restrictions on medication disposal. Changes are needed in policy and practice to address these challenges.

